# Sore throat and obsessions: A causal link?

**DOI:** 10.4103/0019-5545.31608

**Published:** 2006

**Authors:** Chittaranjan Andrade, N Pfizer

**Affiliations:** * Professor Department of Psychopharmacology, National Institute of Mental Health and Neurosciences, Bangalore 560029, Karnataka, India; ** Senior Consultant Department of Psychiatry, Rajah Charitable Medical Trust Hospital, Muthuvattoor, Chavakkad 680506, Kerala, India

Sir,

In 1999, we reported that a 25-year-old woman developed sudden-onset, severe musical obsessions after the experience of an erythromycin-responsive sore throat and fever^1^. We considered that the medical illness was coincidental because of a lack of literature on the relationship between obsessive–compulsive disorder (OCD) phenomena and infections of this nature in adults.^2^

Recent literature has thrown new light on our case. Bodner *et al.*^3^ reported the case of a man who, at the age of 25, also abruptly developed prominent obsessions and compulsions after a severe sore throat. Bodner *et al.*^3^ suggested that the sore throat was responsible for the psychiatric symptoms because, in children, infections with beta-haemolytic streptococci are known to predispose to OCD and movement disorder, a syndrome labelled as Paediatric Autoimmune Neuropsychiatric Disorder Associated with Streptococcus (PANDAS).^4^ The aetiology of PANDAS is unknown; possible causes include an autoimmune antibody or a streptococcal toxin.

PANDAS is by no means a confirmed diagnostic entity. However, Murphy *et al.*^5^ recently helped validate it as a disorder characterized by high streptococcal antibody titers and an episodic or sawtooth course. Symptom exacerbations were associated with elevations in the antibody titers. Tic severity in affected children was greater than that in children with a stable or remitting course of OCD or tic disorder. The tics worsened during the cold seasons, when infections are more common.

What is the risk of PANDAS after a streptococcal infection? Perrin *et al.*^6^ systematically examined the risk of PANDAS in 201 children with a streptococcal sore throat, 207 children with a sore throat of presumed viral aetiology, and 196 well children. PANDAS symptoms were found to be no more common in the streptococcal group than in the other 2 groups after 2 and 12 weeks. There are many possible interpretations of these findings—that the antibiotics which the streptococcal group received protected against PANDAS; that PANDAS is very rare; that, just as there are rheumatogenic streptococcal strains, there may also be ‘PANDAS-genic’ streptococcal strains (if so, the incidence of PANDAS may cluster in seasons of spread of these strains, and may be sparse at other times); or that PANDAS is not a valid diagnostic entity!

How may PANDAS be treated? Murphy and Pichichero^7^ prospectively identified 12 children with new-onset PANDAS. Treatment with antibiotics at the sentinel episode resulted in rapid relief from OCD symptoms. These symptoms recurred in 6 patients; in some, there were multiple recurrences (up to 6) across a 3-year follow up. Each recurrence was associated with a fresh streptococcal infection, and each recurrence responded to antibiotic therapy.

What are the implications of this literature for the case that we reported? We suggest that our patient may have experienced the rare adult form of PANDAS; alternately, the sore throat may have been a trigger for adult-onset OCD. The failure of the obsessions to respond to antibiotics substantiates the trigger hypothesis, unless PANDAS in adults is less antibiotic-responsive.

There is no information about differences in clinical characteristics and clinical course in adult-onset OCD with and without a preceding infection. No data are available on the risk of PANDAS after untreated streptococcal infection in children or adults; the availability of such data might influence policies on antibiotic use for sore throat, especially in children. Swedo *et al.*^4^ proposed criteria for PANDAS in children and adolescents. Criteria for adults remain to be developed. The area clearly requires more research.
